# Three-Dimensional Semantic Segmentation of Diabetic Retinopathy Lesions and Grading Using Transfer Learning

**DOI:** 10.3390/jpm12091454

**Published:** 2022-09-05

**Authors:** Natasha Shaukat, Javeria Amin, Muhammad Sharif, Faisal Azam, Seifedine Kadry, Sujatha Krishnamoorthy

**Affiliations:** 1 Department of Computer Science, COMSATS University Islamabad, Wah Campus, Wah Cantt 47010, Pakistan; 2 Department of Computer Science, University of Wah, Wah Campus, Wah Cantt 47010, Pakistan; 3 Department of Applied Data Science, Noroff University College, 4612 Kristiansand, Norway; 4 Zhejiang Bioinformatics International Science and Technology Cooperation Center, Wenzhou-Kean University, Wenzhou 325060, China; 5 Wenzhou Municipal Key Lab of Applied Biomedical and Biopharmaceutical Informatics, Wenzhou-Kean University, Wenzhou 325060, China

**Keywords:** deeplabv3, convolutional neural network, Messidor, lesions, DR

## Abstract

Diabetic retinopathy (DR) is a drastic disease. DR embarks on vision impairment when it is left undetected. In this article, learning-based techniques are presented for the segmentation and classification of DR lesions. The pre-trained Xception model is utilized for deep feature extraction in the segmentation phase. The extracted features are fed to Deeplabv3 for semantic segmentation. For the training of the segmentation model, an experiment is performed for the selection of the optimal hyperparameters that provided effective segmentation results in the testing phase. The multi-classification model is developed for feature extraction using the fully connected (FC) MatMul layer of efficient-net-b0 and pool-10 of the squeeze-net. The extracted features from both models are fused serially, having the dimension of N × 2020, amidst the best N × 1032 features chosen by applying the marine predictor algorithm (MPA). The multi-classification of the DR lesions into grades 0, 1, 2, and 3 is performed using neural network and KNN classifiers. The proposed method performance is validated on open access datasets such as DIARETDB1, e-ophtha-EX, IDRiD, and Messidor. The obtained results are better compared to those of the latest published works.

## 1. Introduction

**Figure 1:**
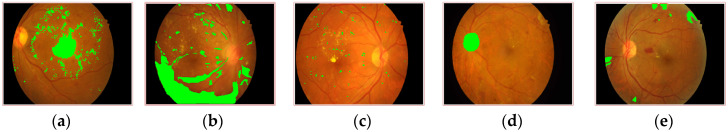
Symptoms of NPDR lesions [9]: (**a**) HEs (**b**) HMs (**c**) MAs (**d**) OD (**e**) SoEX.

Diabetic retinopathy (DR) is the main cause of blindness affecting 93 million people worldwide [1]. It is caused due to microvascular disorders [2,3]. DR is distributed into two classes based on the severity level, proliferative (PDR) and non-proliferative (NPDR). PDR occurs when the retina begins to produce new blood vessels that are more advanced. The emergence of new vessels along the vascular arcades in the retina is usually referred to as neovascularization of the retina [4]. The NPDR is an essential stage of DR. Here, the tiny veins and blood vessels in the retina begin to discharge blood. Its anomalies are categorized according to the severity levels, which are mild, moderate, and severe. Hemorrhages (HMs), hard exudates (HE), soft exudates (SoEX), and microaneurysms (MAs) are common symptoms of NPDR, as shown in Figure 1 [5]. MAs are small red circular dots on the retina created by the damaged vessel walls in the early stage of DR. MAs with prominent edges have a maximum size of 125 micrometers [6]. The blockage of retinal blood vessels causes HMs, which lead to lesions inside the vessels. HMs are divided into two types: flame with surface shape and blot with deep spots [7]. HEs are yellow patches caused by plasma leakage. They have sharp edges and span the outer layers of the retina [8]. SoEX appear as white ovals on the retina due to nerve fiber swelling [6].

Manual DR detection is an error-prone and significant task for an ophthalmologist. Therefore, an automated method is required for precise and rapid detection. In the literature, several computerized methods have been proposed for the detection of DR lesions [10]. Convolutional neural networks (CNNs) and the Hough transform algorithm (HTA) are used for EX detection. Furthermore, histogram equalization and canny edge detection techniques are applied to improve the quality of images. This also avoids inference with the OD, which is an anatomical region. Classification results of 98.53% accuracy on DiaretDB1, 99.17% accuracy on DiaretDB0 and 99.18% accuracy on DrimDB datasets [11] have been achieved. A model based on CNN was developed for DR detection. Multiple preprocessing methods were applied, such as random brightness and random contrast change, which provided an accuracy of 0.92 on the MESSIDOR-2 and 0.958 on the MESSIDOR-1 datasets [12]. Adaptive thresholding also has been used with morphological operators for the segmentation of DR regions. 

Statistical and geometrical features were employed for classification that provided an AUC of 0.99 on E-ophtha and an AUC of 1.00 on Diaretdb, Messidor, and local datasets [13]. The correlated extracted features using two SURF and PHOG descriptors were fused serially using the canonical correlation analysis technique [14]. Although much work has been done in this area, there is still a gap due to the following factors.

Fundus retinal images are used for the analysis of DR. Several challenges exist during the image capturing process, such as illumination noise and poor contrast, that degrade the performance. DR lesion segmentation is also a challenging task due to variable shape, size, and color. Optic disc (OD) detection is another challenge in this domain because it has a circular shape that resembles the retinal lesions. Therefore, it is often falsely detected as a lesion region. To overcome these concerns, a technique is presented for segmenting and classifying the retinal lesions. The contribution steps are discussed as:

(1) The pre-trained Xception model is combined with the Deeplabv3 model. The output of these models is trained on the selected hyperparameters that are finalized after experiments for DR lesion segmentation.
(2) Two transform learning models, efficient-net-b0 and squeeze-net, are employed for feature extraction from the selected fully connected layers such as MATMUL and pool-10, respectively.
(3) The extracted features from MATMUL and pool-10 layers are fused in serial. The adequate features are determined using MPA.
(4) For selection of the best features, the MPA model is trained on the selected hyperparameters. The selected features using the MPA model are passed to the KNN and NN classifiers for DR grade classification.

The article structure is as follows. Section 2 discusses the existing work, Section 3 defines the steps of the proposed method, Section 4 gives results and discussion, and lastly, the conclusion is drafted in Section 5.

## 2. Related Work

Recent work covers the versatile application of conventional and modern approaches for the identification and detection of DR lesions. T-LOP features were used with ELM for classification, providing 99.6% accuracy and 0.991 precision [15]. For detecting HE spots in the blood vessels, a dense, deep feature extraction method based on CNN was proposed, which performs the classification efficiently with an accuracy of 97% and specificity of 92% [16]. Modified Alexnet architecture based on CNN was presented for DR classification. Classification was performed on the MESSIDOR dataset and produced an accuracy of 96% [17]. U-Net residual network with the pre-trained ResNet34 model was presented for the segmentation of DR lesions. The model achieved 99.88% and 0.999% accuracy and dice score, respectively [18]. For classification, a residual-based network architecture was used on the MESSIDOR dataset. Modified models such as ResNet18, ResNet34 and ResNet50 were utilized for binary classification, and obtained an accuracy of 99.47%, 99.47% and 99.87% for ResNet18, ResNet34 and ResNet50, respectively [19]. To improve the segmentation of DR lesions on fundus images, the HEDNet method was proposed. This method claimed that adding adversarial loss enhances the lesion segmentation performance on the IDRiD dataset [20]. A fully connected CNN model was presented with long and short skip connections [2,8,13,21,22,23,24,25,26,27,28,29,30,31,32,33,34,35,36,37,38,39,40,41,42,43,44,45,46,47,48,49,50]. This model was used for segmenting DR lesions, including OD and exudates. Basic FCNs architecture was used for semantic segmentation. For OD segmentation, it obtained sensitivity (SEN) of 93.12% and specificity (Spe) of 99.56%. For exudate segmentation, it achieved Sen of 81.35% and Spe of 98.76% [51]. Using the skip connection in UNet, a deep learning network called MResUNet was proposed for MA segmentation. To solve the pixel imbalance problem between the MA and the background, the authors proposed adaptive weighted loss function cross-entropy on the MResUNet. The proposed network enhanced the performance of network architecture to detect the MA. The MResUNet architecture was evaluated on IDRiD and DiaretDB1 datasets and achieved an SEN of 61.96% on IDRiD and 85.87% on the DiaretDB1 dataset [52]. The pre-trained ResNet-50 and 101 models were used for the classification of DR lesions. They provided an accuracy of 0.9582 on IDRiD, 0.9617 on E-ophtha, and 0.9578 on DDR datasets. Additionally, it was claimed that DARNet beats existing models in terms of robustness and accuracy [53]. The nested U-Net based on the CNN method was proposed for the segmentation of MA and HM and achieved 88.79% SEN on the DIARETDB1 dataset [54]. EAD-Net CNN network architecture was used for EX segmentation with an accuracy of 99.97% [55]. The results were evaluated on seven datasets and achieved an accuracy of 78.6% on DRIVE, 85.1% on DIARETDB1, 83.2% on CHASE-DB1, 80.1% on Shifa, 85.1% on DRIVES-DB1, 87.93% on MESSIDOR, and 86.1% on ONHSD datasets [56].

## 3. Proposed Methodology

**Figure 2:**
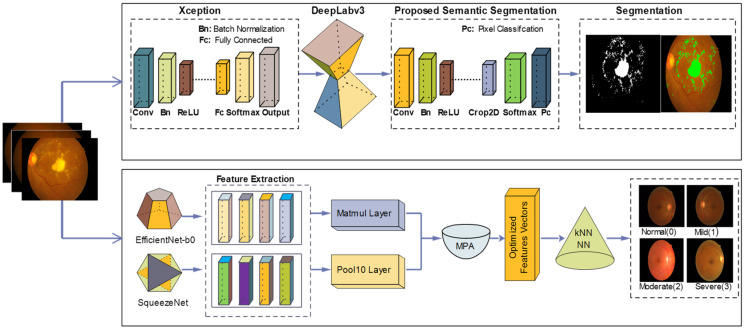
Steps of proposed method for segmentation and classification.

In this research, DR lesion segmentation and classification models are proposed as shown in Figure 2. The proposed segmentation model uses the Xception model [57] with Deeplabv3 on the selected learning parameters. The proposed classification model investigates features using two pre-trained models, i.e., efficient-net-b0 [58] and squeeze-Net [59]. Features extracted from these two models are serially fused and passed on to MPA [60]. Optimal features selected by MPA are fed to the KNN [61] and NN [62] classifiers for DR classification into DR grade 0, 1, 2, and 3.

### 3.1. Proposed Semantic Segmentation Model

**Figure 3:**
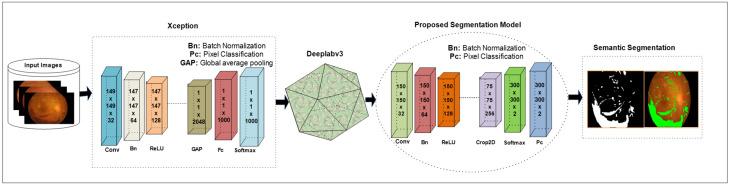
Proposed model for NPDR lesion segmentation.

The DeepLabv3 [63] network is used for segmentation that uses encoder–decoder architecture, skips connection, and dilated convolutions. In this work, the Xception model is used for DR lesion segmentation as an input to the deeplabv3, as shown in Figure 3. The Xception model contains 170 layers, which comprise 1 input, 40 convolutional, 40 batch normalization, 35 ReLU, 34 grouped-convolution, 4 max-pooling, 12 addition, 1 global average pooling, 1 FC, 1 Softmax, and 1 classification output. The proposed segmentation model that is the combination of Xception and Deeplabv3 contains 205 layers, which include 1 input, 48 batch normalization, 49 convolution, 40 grouped convolution, ReLU43, max-pooling 4, addition 12, transposed convolution 2, crop-2D 2, depth concatenation 2, SoftMax, and pixel classification.

The model training is performed based on learning parameters that are chosen after experiments based on the minimum error rate as presented in Table 1.

Table 1 presents the selected hyperparameters for model training, which are selected after experimentation in which Adam optimizer, 200 epochs, 32 batch-sizes, and 0.0001 learning rate provide better results in model testing.

### 3.2. Classification of DR Lesions Using Deep Features

The dominant features are collected from FC layers of the pre-trained efficientNet-b0 and squeeze net models. Efficientnet-b0 consists of 290 layers, which include 1 input, 65 convolutions, 49 batch normalization, 65 sigmoid, 65 element-wise multiplication, 6 grouped convolution, 17 global average pooling, 9 addition, 1 FC softmax, and classification output. The squeezeNet consists of 68 layers, which include 1 output, 26 convolution, 26 ReLU, 3 max-pooling, 8 depth concatenation, 1 drop-out, 1 global average pooling, softmax, and classification output. The Matmul FC layer of efficientnet-b0 and the pool-10 layer of squeezeNet have feature dimensions of N × 1000. These features are fused serially with N × 1000 dimension and fed to the MPA for best feature selection. After MPA best, N × 1032 was selected out of N × 2000 features as an input to the KNN and NN classifiers.

### 3.3. Feature Selection Using MPA

This research employs MPA for feature selection, where N × 1032 optimal features are selected out of N × 2000, which provide better results for the classification.

MPA is an optimization algorithm that is built up using the population of particles. The survival of particles is determined by the fittest hypothesis. It comprises three distinctive optimization scenarios depending on velocity ratio (v). The high-velocity proportion (v ≥ 10) shows that the prey successfully outruns the predators by using the high-velocity extent.

The low-velocity proportion (v = 0.1) shows that predators can invade the prey. Here, the predator adopts the Levy development strategy. MH algorithms deliver the basic populace of self-assertive look specialists depending on prior information. At this point, the MPA algorithm updates self-assertive look agent zones in all accentuation and at last obtains the finest ideal solution depending on the optimization issue. Equation (1) considers z as an arbitrary search operator extending over the interval z ∈ [lb, ub], and $\bar{z}$ is an opposite search operator.


(1) $$\bar{z}=\mathrm{lb}+\mathrm{ub}-z$$

In the above equation, ‘lb’ and ‘ub’ indicate lower-bound and upper-bound arbitrary search agents respectively. Equations (2) and (3) show the arbitrary search operator created in ‘n’ dimension search spaces.

z = [z_1_, z_2_, z_3_, …. z_n_] (2)

(3) $$\bar{z}=[{\bar{z}}_{1},{\bar{z}}_{2},\bar{z},\ldots {\bar{z}}_{n}]$$

In Equation (4), opposite values $\bar{z}$ are created

(4) $${\bar{z}}_{j}=lb_{j}+ub_{j}-z_{j}$$

In Equation (5), Φ represents the stability estimator; it is used to measure the distinctive cardinality of feature sets and determine the stability of the model.

(5) $$\;\Phi \left(B\right)=1-\frac{\frac{1}{x}\sum_{h=1\;}^{x}S^{2}{}_{h}}{\frac{\bar{N}}{x}\left(\;1-\frac{\bar{N}}{x}\right)}$$

where

(6) $$S^{2}{}_{h}\;=\frac{W}{W-1}{\hat{r}}_{h}\left(1-{\hat{r}}_{h}\right)$$

and

(7) $$\bar{N}=\sum_{h=1}^{x}\;\frac{1}{W}\cdot \sum_{i=1}^{W}{\;Y}_{i,h}$$

In Equation (6), W represents the rows in a binary matrix (B), and ${\hat{r}}_{h}$ indicates the recurrence of the specified features that are selected at the time of iteration operation. In Equation (7), $\bar{N}$ represents the average features chosen in binary matrix and $Y_{i,h}$ indicates the binary value in ‘ith’ row and ‘hth’ column. The pre-trained values of MPA are used for best feature selections as shown in Table 2.

**Figure 4:**
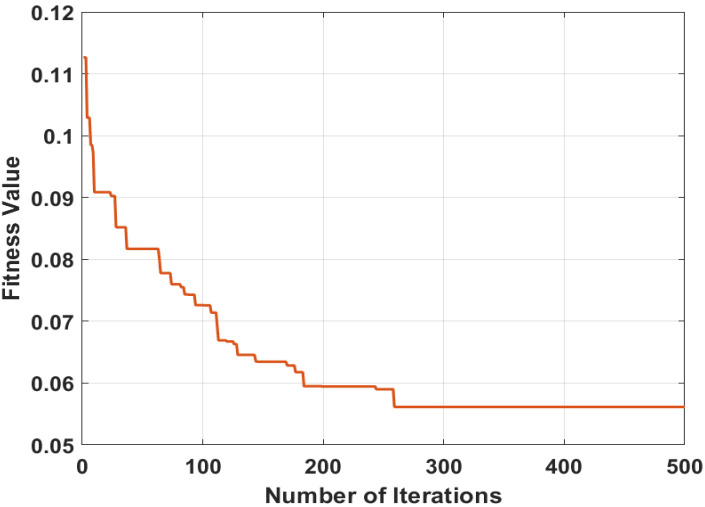
Best selected features using MPA.

Here, lb is 0, ub is 1, thres is 0.5, beta is 1.5, P is 0.5 and FADs is 0.2; by using these parameters of MPA classification, the error rate is minimized, which gives better results. The graphical representation of MPA is depicted in Figure 4.

The conversion plot of MPA between the fitness value and the number of iterations is shown in Figure 4. Here, the plotted curve identifies the error rate, which is constant after 300 iterations.

## 4. Experimental Discussion

The publicly available MESSIDOR dataset is used for DR classification. The dataset contains 1200 color eye fundus images of each class. These images are provided in three sets belonging to different ophthalmologic departments. Each image set has four zipped subsets containing 100 images in TIFF format. Flip, horizontal and vertical augmentation are applied to this dataset to balance the number of images. Augmentation applied at each level of the MESSIDOR dataset is listed hereunder.

(1) Grade0 = 1092 images
(2) Grade1 = 1224 images
(3) Grade2 = 1976 images
(4) Grade3 = 1016 images

The above-mentioned 5308 augmented images are used to avoid the overfitting problem. These images are captured from a 3CCD camera with a view of 45 degrees and divided into four classes [9]. The detail of the classification dataset is shown in Table 3. In this research, segmentation datasets IDRID, DIARETDB1, and e-ophtha-EX are used [64]. Forty-seven images are of e-ophtha-EX, out of which 35 are healthy [65]. IDRiD contains 81 MA, 81 EX, 80 HE, and 40 SoEX [66].

Table 3 presents the description of the publicly available Messidor dataset, which is used for classification. The summary of the segmentation datasets is mentioned in Table 4.

MATLAB 2021RA with a Core-i7 CPU, 8 GB Nvidia graphic card 2070 RTX, and 32 GB RAM are used for the implementation of this research.

### 4.1. Experiment 1: DR-Lesions Segmentation

The semantic segmentation method is used to segment the multi-class DR lesions such as MA, HM, HE, SE, and OD. Here, the model is trained with the ground truth mask that gives the best result in the testing phase. It uses mIoU, mDice, F1-score, precision, recall, and accuracy measures as presented in Table 5.

**Figure 5:**
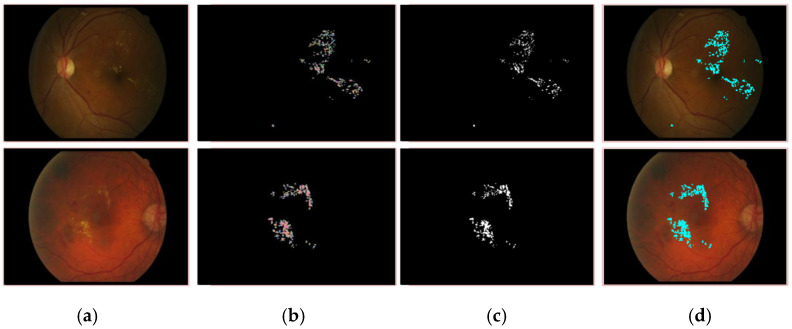
Segmented EX lesions of the e-ophtha-EX dataset. (**a**) Input image, (**b**) proposed segmentation, (**c**) ground truth image, (**d**) segmented region mapped on the input image.

**Figure 6:**
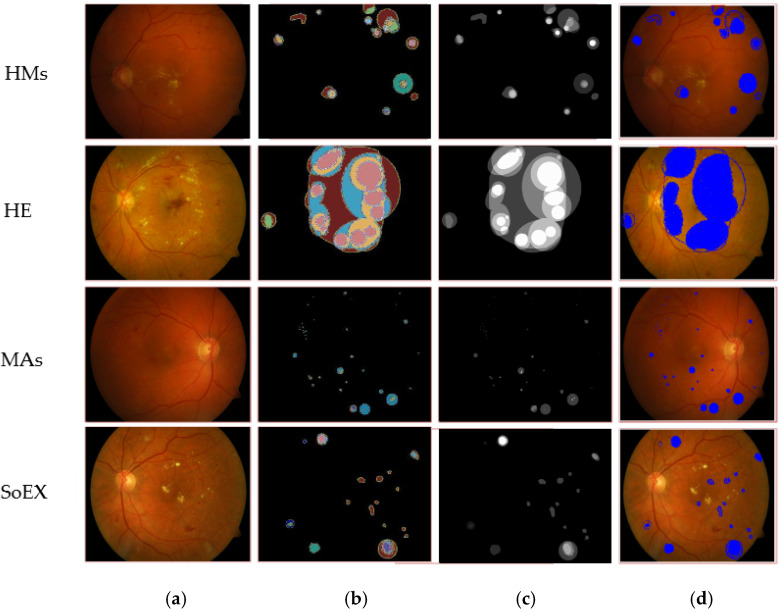
Segmented DR lesion outcomes on DIARETDB1 dataset. (**a**) Input image, (**b**) proposed segmentation, (**c**) ground truth image, (**d**) segmented region mapped on the input image.

**Figure 7:**
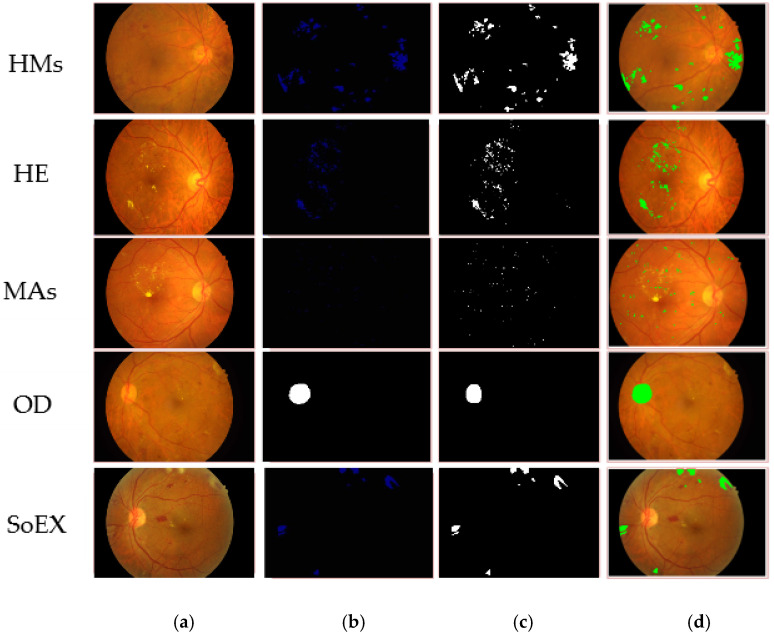
Segmented DR lesions on IDRiD dataset. (**a**) Input image, (**b**) proposed segmentation, (**c**) ground truth image, (**d**) segmented region mapped on the input image.

Table 5 shows the segmentation results on three benchmark datasets such as e-ophtha-EX, DIARETDB1, and IDRiD. The proposed model gives mIoU of 0.94 for EX; on DIARETDB1, 0.87 mIoU for HM, 0.71 mIoU for HE, 0.87 mIoU for MA, and 0.86 mIoU for SoEX; on the IDRiD dataset, 0.86 mIoU for HM, 0.88 mIoU for HE, 0.71 mIoU for MA, 0.86 mIoU for OD, and 0.84 mIoU for SoEX. The proposed method segmentation results for the benchmark datasets are given in Figure 5, Figure 6 and Figure 7.

Table 6 compares the results of the segmentation approach with the existing methods.

Table 6 presents the results for segmentation of the DR lesions using IDRiD, E-ophtha, and DIARETDB1 datasets. DARNet is proposed for segmentation using IDRiD and e-ophtha-EX datasets that provide an average accuracy of 0.9582 on IDRiD and 0.9617 on e-ophtha-EX [53]. A nested U-Net Zhou is used for red lesion segmentation using the DIARETDB1 dataset, which provides 79.21% F1-Score and 88.79% SEN [54]. EAD-Net architecture is presented for the segmentation using the e-ophtha-EX dataset. A modified U-Net network is used for DR lesion segmentation, such as for MA and HE. It is used on IDRiD and e-ophtha datasets. This network obtains 99.88% accuracy and a 0.9998 dice score both for MA and HE segmentation [18]. The MResUNet model is used for MA segmentation. The model achieves SEN of 61.96% for IDRID and 85.87% for DiaretDB1 datasets [52].

The existing methods are evaluated in terms of average accuracy; therefore, the proposed method results are compared to the existing methods using average accuracy measures on three benchmark datasets such as IDRiD, E-ophtha, and DIARETDB1.

The results presented in Table 6 show that the proposed method provides 0.96 Acc and 0.98 Sensitivity on the IDRiD dataset, while the existing methods [53,67,68,69] yield 0.95 Acc, 0.87 Sensitivity, 0.83 Acc, and 0.80 Acc, respectively.

Similarly, the proposed method performed better on the E-ophtha dataset, having an Acc of 0.97, which is greater than that of the existing method [53] with an Acc of 0.96.

The proposed method also provided better results on the DIARETDB1 dataset with 0.99 Sensitivity as compared to existing methods [52,54,70] that provided 0.88, 0.61, and 0.85 sensitivity, respectively. It is concluded that the overall proposed method performed well on three benchmark datasets such as IDRiD, E-ophtha, and DIARETDB1 as compared to all existing methods having the same datasets and average accuracy measure.

In comparison to the previous works, the segmentation model in this research is developed by combining Xception and Deeplabv3 models. These models are trained on optimal hyperparameters that provide improved segmentation results.

### 4.2. Experiment 2: DR Lesions Classification

**Figure 8:**
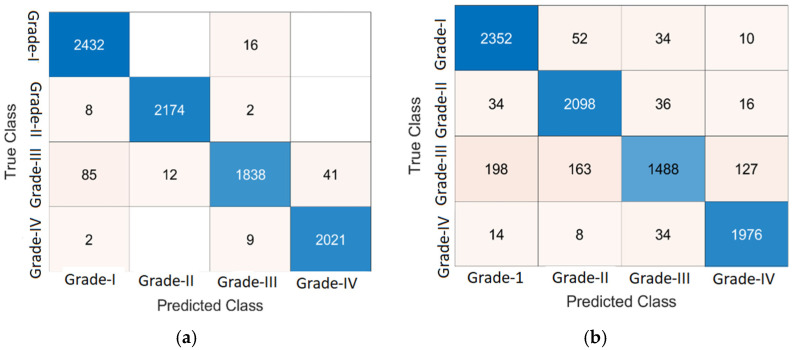
Classification of DR lesions. (**a**) KNN of the optimizable kernel, (**b**) NN of medium neural network.

In this experiment, two benchmark classifiers, KNN and NN, are used for DR classification. The DR classification results are computed on a benchmark dataset as presented in Table 7 and Table 8. Figure 8 presents the classification results in a confusion matrix.

Figure 8 presents the results of classification on 10-fold cross-validation using KNN and NN classifiers. The computed classification results are presented in Table 7 using a KNN classifier.

Table 7 shows the classification result of KNN on 10-fold cross-validation. Here, the grade 0 level achieves 86.57% accuracy by using weighted KNN, 98.72% by optimizable KNN, 96.75% by cosine KNN, and 97.94% by fine KNN. Grade 1 yields the following accuracies: 97.08% by weighted KNN, 99.75% by optimizable KNN, 95.56% by cosine KNN, and 99.29%by fine KNN. The attained accuracy for grade 2 is 87.77% using weighted KNN, 98.09% by optimizable KNN, 96.53% by cosine KNN, and 97.73% by fine KNN. Achieved accuracy in grade 3 is 98.32% by using weighted KNN, 99.4% by optimizable KNN, 98.48% by cosine KNN, and 99.29% by fine KNN. The DR classification results of the NN classifier on 10-fold cross-validation are presented in Table 8.

Table 8 shows the classification results of NN on 10-fold cross-validation. Here, grade 0 gives 95.8% accuracy by using a narrow neural network, 95.78% by medium neural network, 96.04% by wide neural network, 95.35% by bilayered neural network, and 94.12% by trilayered neural network. In grade 1, the following accuracies are achieved: 95.67% by narrow neural network, 96.38% by medium neural network, 96.42% by wide neural network, 95.56% by bilayered neural network, and 95.34% by trilayered neural network. The attained accuracy for grade 2 is 90.94% by narrow neural network, 92.11% by medium neural network, 93.15% by wide neural network, 90.47% by bilayered neural network, and 89.04% by trilayered neural network. Achieved accuracies in grade 3 are 96.2% by narrow neural network, 97.04% by medium neural network, 97.58% by wide neural network, 96.35% by bilayered neural network, and 96.16% by trilayered neural network.

### 4.3. Significance Test

**Figure 9:**
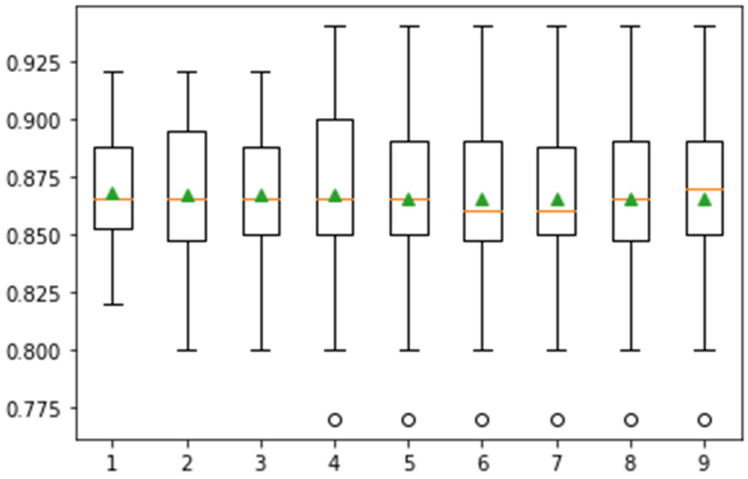
Confidence interval of classification accuracy by performing Monte Carlo simulation on 10-fold cross-validation using Messidor dataset.

In this experiment, Monte Carlo simulation is performed using fine KNN on the Messidor dataset. Here, mean and standard deviation values are computed using 10, 15, and 20 different iterations. The classification results of these iterations, including the mean and standard deviation of classification with a graphical representation of the classification score, are presented in Figure 9.

**Figure 10:**
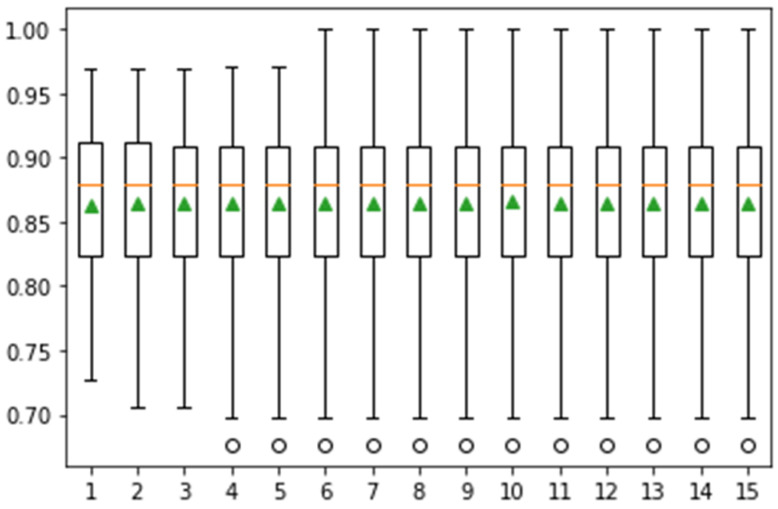
Confidence interval of classification accuracy by performing Monte Carlo simulation on 15 iterations using Messidor dataset.

In Figure 9, the whisker box plot represents the distribution of the score. Here, the orange color represents the median distribution, and the triangle green color shows the arithmetic mean. Distribution is symmetric on the same symbol values, and the mean value attain the central position. This method provides help for the selection of appropriate heuristic values using different iterations. On 10-fold cross-validation, greater than 0.925 accuracy is achieved. The classification task is performed in 15-fold, and the results are presented in Figure 10.

**Figure 11:**
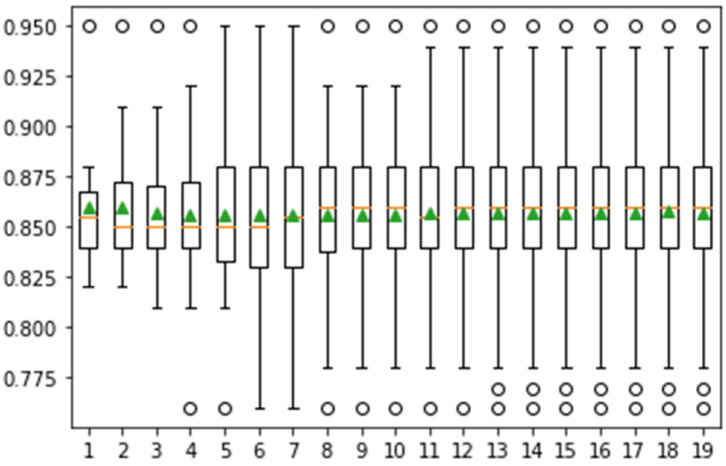
Confidence interval of classification accuracy by performing Monte Carlo simulation on 20 iterations using Messidor dataset.

In Figure 10, the achieved classification accuracy is greater than 0.95. The classification results are computed on 20 iterations, as depicted in Figure 11.

The classification accuracy after 20 cross-fold validation is greater than 0.95. Table 9 presents the comparison results of the classification methods.

VGG-16 and Inception-V3 provide 98.5%, 98.9%, and 98.0% accuracy, SEN, and specificity, respectively [71]. For classification, Alexnet architecture gives the accuracy of 96.6% on grade 0, 96.2% on grade 1, 95.6% on grade 2 and 96.6% on grade 3 [17]. Different architectures are used for the classification of DR. Optimal results are obtained using ResNet50-based architecture with achieved accuracy of 0.92 for grade 0 and grade 1, and 0.93 and 0.81 for grade 2 and grade 3, respectively [1]. A capsule network is proposed for classification of DR lesions that provides accuracy of 97.98% for grade 0, 97.65% for grade 1, 97.65% for grade 2, and 98.64% for grade 3 [72]. The pre-trained Inception-ResNet-v2 model is used for classification that provides an accuracy of 72.33% on the MESSIDOR dataset [73]. The proposed classification model achieves better results because of serially fused features and optimum feature selection by MPA optimizer.

## 5. Conclusions

DR lesion segmentation and classification are challenging tasks. Some challenges include variable size shape and irregular location of the lesion. The Xception and deeplabv3 models have been integrated to devise a single model. This model is trained on selected hyperparameters for DR lesion segmentation. The proposed model optimally segments the DR lesions. The performance measures are 0.94 for mIoU, 0.97 for mDice, 0.99 for F1-score, 0.95 for precision, and 0.99 for Recall on the e-ophtha-EX dataset. Further, performance on the DIARETDB1 dataset includes mIOU of 0.87 on HM, 0.71 on HE, 0.87 on MA, and 0.86 on SoEX. Using the IDRiD dataset, mIOU of 0.86 on HM, 0.88 on HE, 0.71 on MA, 0.86 on OD, and 0.84 on SoEX are achieved. The classification is performed based on 10-fold cross-validation using KNN and neural network classifiers. Optimizable KNN achieved an accuracy of 97.97%, with a precision of 0.99 on grade 0, 1.00 on grade 1, 0.93 on grade 2, and 0.99 on grade 3. The proposed classification model accurately classifies the DR grades using serially fused features and a swarm intelligence algorithm.

## Figures and Tables

**Table 1:** Hyperparameters of the proposed segmentation model.

Optimizer	Epochs for Training	Size Batch	Learning Rate	Error Rate
**Sgdm**	**200**	32	**0.0001**	**0.02**
Adam		16		0.04
RmsProp		8		0.06
Momentum		64		0.07

**Table 2:** Pre-trained parameters of MPA.

Lower Bound (lb)	0
Upper Bound (ub)	1
Threshold (thres)	0.5
Levy Component (beta)	1.5
Constant (P)	0.5
Fish Aggregating Devices Effect (FADs)	0.2

**Table 3:** Classification dataset description.

DR Levels	Description	Grades
Normal	Healthy	0
Mild (NPDR)	Mas	1
Moderate (NPDR)	Few MAs or HMs	2
PDR	More MAs and HMs	3

**Table 4:** Description of segmentation datasets.

Datasets	Description
DIARETDB1	Total images:89 Normal:5 Mild NPDR:84
e-ophtha-EX	Normal: 35 Exudates:47
IDRiD	MAs:81 HE:81 HMs:80 SoEX:40

**Table 5:** Results of segmentation method using benchmark datasets.

Datasets	Lesions	mIoU	mDice	F1-Score	Precision (P)	Recall	Accuracy (Acc)
**e-ophtha-EX**	EX	0.94	0.97	0.98	0.94	0.99	0.96
**DIARETDB1**	HM	0.87	0.83	0.72	0.87	0.99	0.87
	HE	0.71	0.83	0.92	0.71	0.99	0.71
	MA	0.87	0.83	0.72	0.87	0.99	0.87
	SE	0.86	0.88	0.87	0.86	1.00	0.86
**IDRiD**	HM	0.86	0.88	0.88	0.86	1.00	0.86
	HE	0.88	0.84	0.81	0.88	1.00	0.88
	MA	0.71	0.83	0.92	0.71	1.00	0.71
	OD	0.86	0.87	0.87	0.86	1.00	0.86
	SE	0.84	0.83	0.82	0.87	0.98	0.97

**Table 6:** Comparison of segmentation results.

Ref	Year	Method	Dataset	Results
[53]	2022	Dual-input attentive RefineNet (DARNet)	IDRiD	0.95 Acc
[67]	2021	U-Net		0.87 Sensitivity
[68]	2021	U-Net		0.83 Acc
[69]	2021	SVM		0.80 Acc
**Proposed Model**				**0.96 Acc, 0.98 Sensitivity**
[53]	2022	DARNet	E-ophtha-EX	0.96 Acc
**Proposed Model**				**0.97 Acc**
[54]	2022	Nested U-Net	DIARETDB1	0.88 Sensitivity
[52]	2021	MResUNet		0.61 Sensitivity
[70]	2020	U-Net		0.85 Sensitivity
**Proposed Model**				**0.99 Sensitivity**

**Table 7:** Classification results on 10-fold cross-validation using KNN classifier.

	Grade	Accuracy	Precision	Recall	F1 Score	Overall Accuracy
**Weighted KNN**	0	86.57%	1.00	0.68	0.81	84.87%
	1	97.08%	0.94	0.94	0.94	
	2	87.77%	0.47	1.00	0.64	
	3	98.32%	0.94	0.99	0.96	
**Optimizable KNN**	0	98.72%	0.99	0.96	0.98	97.97%
	1	99.75%	1.00	0.99	0.99	
	2	98.09%	0.93	0.99	0.96	
	3	99.40%	0.99	0.98	0.99	
**Cosine KNN**	0	96.75%	0.99	0.91	0.95	93.66%
	1	95.56%	0.84	0.99	0.90	
	2	96.53%	0.94	0.91	0.93	
	3	98.48%	0.98	0.96	0.97	
**Fine KNN**	0	97.94%	1.00	0.94	0.96	97.13%
	1	99.29%	0.99	0.98	0.99	
	2	97.73%	0.91	0.99	0.95	
	3	99.29%	0.98	0.99	0.98	

**Table 8:** Classification results on 10-fold cross-validation using NN classifier.

**Narrow Neural Network**	**Grade**	**Accuracy**	**Precision**	**Recall**	**F1 Score**	**Overall Accuracy**
	0	95.80%	0.96	0.90	0.93	89.31%
	1	95.67%	0.96	0.88	0.92	
	2	90.94%	0.67	0.91	0.77	
	3	96.20%	0.97	0.88	0.92	
**Medium Neural Network**	0	95.78%	0.96	0.90	0.93	90.65%
	1	96.38%	0.96	0.91	0.93	
	2	92.11%	0.72	0.92	0.81	
	3	97.04%	0.97	0.91	0.94	
**Wide Neural Network**	0	96.04%	0.96	0.91	0.93	91.60%
	1	96.42%	0.96	0.90	0.93	
	2	93.15%	0.75	0.93	0.83	
	3	97.58%	0.97	0.93	0.95	
**Bilayered Neural Network**	0	95.35%	0.95	0.89	0.92	88.87%
	1	95.56%	0.95	0.88	0.92	
	2	90.47%	0.67	0.89	0.76	
	3	96.35%	0.96	0.89	0.93	
**Trilayered Neural Network**	0	94.12%	0.94	0.86	0.90	87.33%
	1	95.34%	0.95	0.88	0.91	
	2	89.04%	0.61	0.87	0.72	
	3	96.16%	0.96	0.89	0.92	

**Table 9:** Comparison of classification results.

Ref#	Year	Method	Dataset	Overall Acc%	Grade-0%	Grade-1%	Grade-2%	Grade-3%
[71]	2022	WFDLN	Messidor	0.98	-	-	-	-
[17]	2019	Modified Alexnet		-	0.96	0.96	0.95	0.96
[1]	2021	ResNet50		-	0.93	0.93	0.81	0.92
[72]	2021	CapsNet		-	0.97	0.97	0.97	0.98
[73]	2021	Inception-ResNet-v2		0.72	-	-	-	-
**Proposed method**				0.99	0.98	0.99	0.98	0.99
